# Identification of Prognostic Genes Relevant With the Nuclear Factors of Activated T Cells Based on Transcriptomics in Oral Squamous Cell Carcinoma

**DOI:** 10.1002/cre2.70408

**Published:** 2026-07-19

**Authors:** Julaiti Tuerxun, Ailimaierdan Ainiwaer, Tairan Ding

**Affiliations:** ^1^ Department of Oral and Maxillofacial Trauma and Orthognathic Surgery The First Affiliated Hospital of Xinjiang Medical University, Research Institute of Stomatology of Xinjiang Uygur Autonomous Region Urumqi China; ^2^ Outpatient Department of Oral Surgery The First Affiliated Hospital of Xinjiang Medical University, Research Institute of Stomatology of Xinjiang Uygur Autonomous Region Urumqi China

**Keywords:** oral squamous cell carcinoma, prognostic genes, risk score, tumor mutational burden

## Abstract

**Background:**

It has previously been demonstrated that the nuclear factor of activated T cells (NFAT) is crucial for the development of tumors. Given OSCC's drug resistance and poor outcomes, identifying NFAT‐associated prognostic genes is urgent for better treatment.

**Material and Methods:**

The TCGA‐OSCC and GSE41613 datasets were utilized to identify differentially expressed genes (DEGs) between oral squamous cell carcinoma (OSCC) and controls. Specifically, differentially expressed genes related to NFAT (DEG‐NFATs) were further screened for NFAT scores in OSCC versus control. The intersection of DEGs and DEG‐NFATs was taken to identify differentially expressed NFAT‐related genes (DE‐NFATRGs). Univariate Cox and least absolute shrinkage and selection operator (LASSO) regression analyses were employed to identify prognostic genes. A risk score was developed based solely on the expression levels of the seven‐gene signature. Subsequently, independent prognostic analyses incorporating clinicopathological variables were performed using univariate and multivariate Cox regression to evaluate whether the risk score served as an independent predictor of survival. Lastly, targeted therapeutic agents for OSCC were predicted. In addition, prognostic gene expression was validated using reverse transcription‐quantitative polymerase chain reaction (RT‐qPCR).

**Results:**

Totally 4463 DEGs obtained were intersected with 310 DEG‐NFATs to obtain 263 DE‐NFATRGs. A risk model can be built using the seven prognostic genes associated with NFAT (ALB, PDK4, SERPINA5, SPINK6, SERPINA9, CGNL1 and IL17F). The risk score accurately predicts survival in OSCC patients. Finally, a total of 31 drugs with significant differences were predicted between risk groups. The most significant of these were AG.014699, Midostaurin, Gefitinib, and LFM.A13. Besides, the expression levels of ALB, PDK4, and SERPINA5 were significantly higher in tumors, while the expression levels of CGNL1 and IL17F were significantly lower in tumors.

**Conclusion:**

The identification of new prognostic genes (ALB, PDK4, SERPINA5, SPINK6, SERPINA9, CGNL1, and IL17F) was provided for the prognosis and treatment of OSCC.

**Abbreviations**
APC: activated protein C
CAR: C‐reactive protein‐to‐albumin ratio
CFB: complement factor B
CSCs: cancer stem cells
DADA: diisopropylamine dichloroacetate
DEGs: differentially expressed genes
DEG‐NFATs: differentially expressed genes of NFAT
DE‐NFATRGs: differentially expressed NFAT‐related genes
EGFR: epidermal growth factor receptor
EMT: epithelial‐mesenchymal transition
ESCC: esophageal squamous cell carcinoma
GDSC: Genomics of Drug Sensitivity in Cancer
GEO: Gene Expression Omnibus
GO: Gene Ontology
GSEA: gene set enrichment analysis
HCM: hypertrophic cardiomyopathy
HGBC: high‐grade bladder urothelial carcinoma
HNSCC: head and neck squamous cell carcinoma
HPV: human papillomavirus
IL‐2: interleukin‐2
KEGG: Kyoto Encyclopedia of Genes and Genomes
LASSO: least absolute shrinkage and selection operator
NFAT: nuclear factor of activated T
NFATRGs: NFAT‐related genes
NK: natural killer
NPAR: Neutrophil percentage‐to‐albumin ratio
OL: oral leukoplakia
OPMDs: oral potentially malignant disorders
OSCC: oral squamous cell carcinoma
PDH: pyruvate dehydrogenase
PDKs: pyruvate dehydrogenase kinases
PLAU: pro‐fibrinolytic
ROC: receiver operating characteristic
ROS: reactive oxygen species
TIDE: Tumor Immune Dysfunction and Exclusion
TMB: tumor mutational load
TME: tumor microenvironment
VEGF: vascular endothelial growth factor

## 1 Introduction

According to IARC data, lip and oral cavity cancers showed significant global incidence and mortality in 2020 (Sung et al. 2021). Oral squamous cell carcinoma (OSCC), the predominant head and neck squamous cell carcinoma subtype, represents a major health burden (Ferlay et al. 2021). Disease development is primarily linked to alcohol consumption and human papillomavirus infection (Boccia et al. 2009; Wu et al. 2018). Standard treatments—surgery, radiation, and chemotherapy—offer limited efficacy with severe side effects in advanced disease (Niu et al. 2022; Siegel et al. 2023). While targeted therapies have shown promise (Liu, Chen et al. 2019), OSCC remains a multifactorial malignancy with poor prognosis, and the lack of effective early diagnostics and biomarkers underscores the urgent need for personalized strategies (Chen et al. 2019; Kaur et al. 2018).

T cells play a pivotal role in cancer immunotherapy, with T cell infiltration serving as a key prognostic factor (Fridman et al. 2012; Maibach et al. 2020). The Nuclear Factor of Activated T cells (NFAT) family of transcription factors regulates T cell proliferation and function primarily through promoting interleukin‐2 (IL‐2) and its receptor expression. Furthermore, NFAT proteins participate in diverse immune responses and exhibit distinct functional effects across T cell subsets (Chen et al. 1999; Macian 2005; Shaw et al. 1988), while also maintaining diverse regulatory functions in multiple physiological systems (Canalis et al. 2022; Weider et al. 2018). Concurrently, NFAT signaling plays multifaceted roles in tumorigenesis, including regulation of tumor angiogenesis (Aurora et al. 2010; Courtwright et al. 2009), cell proliferation and metastasis, and metabolic reprogramming (Jiang et al. 2019; Lehen'Kyi et al. 2007; Liu, Liang et al. 2019; Pan et al. 2013; Singh et al. 2010). In OSCC, emerging evidence indicates that NFAT members such as NFATc3 and NFAT5 promote disease progression through mechanisms involving cancer stem cell regulation and epidermal growth factor receptor (EGFR) membrane trafficking (Lee et al. 2016; Nguyen et al. 2022; Yoshimoto et al. 2021). However, despite the growing recognition of NFAT pathway significance, there remains a notable absence of studies systematically developing and validating OSCC prognostic models based on NFAT‐associated gene signatures. The identification of key prognostic genes closely associated with NFAT signaling would provide valuable insights into OSCC molecular mechanisms and enhance risk stratification for patients.

Given the escalating incidence and complex etiology of OSCC, this study aims to identify prognostic biomarkers closely associated with the NFAT signaling cascade by integrating transcriptomic data and bioinformatics analyses. We hypothesize that a distinct set of NFAT‐related genes exists, whose expression profiles can accurately predict clinical outcomes in OSCC patients. To test this hypothesis, we will delineate NFAT‐associated genes demonstrating differential expression in OSCC, then construct and validate a prognostic risk model based on these biomarkers. Furthermore, we will investigate the correlation between this risk model and the tumor immune microenvironment, as well as its association with sensitivity to targeted therapeutic agents. This research is expected to provide novel biomarkers and theoretical foundations for precision prognosis and treatment of OSCC, thereby enabling clinicians to implement more aggressive therapeutic strategies and ultimately improve patient survival rates and quality of life.

## 2 Materials and Methods

### 2.1 Data Source

Transcriptome sequencing data and corresponding clinical information for OSCC were obtained from The Cancer Genome Atlas (TCGA) database (https://portal.gdc.cancer.gov/). To construct a disease‐specific cohort, we extracted data from samples originating from the oral cavity, floor of the mouth, jaw, and buccal mucosa. The final curated TCGA‐OSCC dataset comprised 260 OSCC tumor samples and 19 adjacent normal tissue samples, which served as controls. The Gene Expression Omnibus (GEO) database (https://www.ncbi.nlm.nih.gov/geo/) was accessed, and the GSE41613 acquired for risk model validation, which contained 97 patients with complete transcriptome sequencing data of OSCC patients (Zhao et al. 2022). The set of transcription factor‐related genes of the nuclear factor of activated T cells (NFAT) was extracted from previous research, containing 46 NFAT‐related genes (NFATRGs) (Gabriel et al. 2016).

### 2.2 Discrimination of Differentially Expressed NFATRGs (DE‐NFATRGs)

First, differentially expressed genes (DEGs) between OSCC and controls in the TCGA‐OSCC dataset were screened with the program “DESeq. 2” (version 1.38.0) (| log2FC | > 1, *p*‐value < 0.05) (Li et al. 2022; Love 2014). Then, the “ggplot2” (version 3.4.1) and the “pheatmap” (version 2.14.0) were utilized to plot volcano maps and heatmaps, respectively (Song et al. 2022; Steenwyk and Rokas 2021). NFAT pathway activity was evaluated using the ssGSEA algorithm in the GSVA package (v1.42.0) with a 46‐gene NFAT signature (Gabriel et al. 2016). Input data comprised log2‐transformed FPKM‐normalized expression matrices, analyzed via Poisson kernel (kcdf = “Poisson”) and ssgsea.norm = TRUE for sample comparability. OSCC samples were stratified into high‐ (score ≥ median, *n* = 139) and low‐NFAT (score < median, *n* = 140) groups. Differential expression between groups was performed using DESeq. 2 (v1.38.0) with model ~condition (High vs. Low), identifying DEG‐NFATs at |log2FC| > 1 and *p*.adj < 0.05 (Li et al. 2022). Finally, DEG‐NFATs were intersected with OSCC‐dysregulated DEGs (|log2FC|> 1, *p*.adj < 0.05).

### 2.3 Enrichment Analysis of DE‐NFATRGs and Creation of Protein‐Protein Interaction (PPI) Networks

In order to explore the biological functions and signaling pathways involved in the DE‐NFATRGs, Gene Ontology (GO) and Kyoto Encyclopedia of Genes and Genomes (KEGG) enrichment analysis of DE‐NFATRGs were conducted using the “clusterProfiler” (version 4.2.2) (Wu 2021), with a screening requirement of *p*‐value < 0.05. In order to explore the interaction of DE‐NFATRGs at the protein level, a PPI network of DE‐NFATRGs was established based on the STRING database (http://www/string-db.org/) (interaction score > 0.9) and utilized the “Cytoscape” (version 3.8.2) for network mapping (Shannon 2003).

### 2.4 Discrimination of the NFAT‐Related Prognostic Genes

Univariate Cox was first carried out on DE‐NFATRGs using the “survival” (version 3.5‐3) to screen out genes that significantly associated with survival of OSCC patients (HR ≠ 1, *p*‐value < 0.05) as NFAT‐related prognostic genes (Fu and Gilbert 2017). NFAT‐related prognostic genes were identified by least absolute shrinkage and selection operator (LASSO) analysis applying “glmnet” (version 4.1‐4) (Friedman et al. 2010).

### 2.5 Creation and Confirmation of the Risk Model

A risk model was ultimately constructed based on the expression levels of NFAT‐related prognostic genes and their corresponding LASSO regression coefficients. First, the risk score for each OSCC tumor sample in the TCGA‑OSCC dataset was calculated using the LASSO‑derived coefficient for each prognostic gene (denoted as CoefficientGene i) and its expression level in the sample (denoted as ExpressionGene i). Subsequently, patients with OSCC in the dataset were divided into high‐ and low‐risk groups according to their risk scores. Similarly, the risk model was validated in the GSE41613 dataset following the same procedure. Kaplan–Meier (K–M) survival curves were then plotted using the “survminer” (version 0.4.9), and differences in survival rates between groups were evaluated with the log‑rank test (significance threshold *p*‐value < 0.05) (Liu et al. 2021). Finally, receiver operating characteristic (ROC) curves were generated to assess the utility of the risk model in predicting the prognosis OSCC. The area under the curve (AUC) was calculated to evaluate the accuracy of the model in predicting 1‑, 2‑, and 3‑year survival rates (AUC > 0.6 was considered acceptable). The “pROC” package (version 1.18.0) was used for plotting (Robin 2011), and the specific workflow is shown in Figure S1. The risk score was calculated as follows:

$$\text{Risk score}\;=\sum_{i=1}^{n}\mathrm{Expression}\;\mathrm{Gene}\;i\times \mathrm{Coefficient}\;\mathrm{Gene}\;i$$

### 2.6 Independent Prognostic Analysis

To explore differences in risk scores among different pathological feature groups, First, risk scores were compared between different pathological feature groups (such as age, TNM stage, and gender) in the TCGA‐OSCC dataset by the Wilcoxon test. Independent prognostic factors for patient survival prediction were then constructed based on univariate Cox regression analysis, combining risk scores with different pathologic characteristics (*p*‐value < 0.05). Next, pathologic features screened by the last step were used in multivariate Cox regression analysis by the PH assumption test (*p*‐value > 0.05) to identify independent prognostic factors. The “rms” (version 6.7‐0) was utilized to construct a nomogram model and calibration curves, and the clinical utility of the nomogram was assessed using decision curve analysis (DCA) (Lai et al. 2022).

### 2.7 Gene Set Enrichment Analysis (GSEA)

Differential expression analysis was performed via the R package “DESeq. 2” on the training set high‐/low‐risk groups to derive log2FC, then genes were ranked by decreasing log2FC. Subsequent GSEA used clusterProfiler with MSigDB KEGG set “c2.kegg.v7.4.symbols” as reference, with |NES | > 1 and *p*‐value < 0.05 as significance thresholds (Wen et al. 2025).

### 2.8 Immune Infiltration Analysis

To validate the specific association between GSEA‐enriched pathways and the tumor microenvironment, the proportion of 22 immune cells in the TCGA‐OSCC dataset was first determined using CIBERSORT. Next, the Wilcoxon test was accepted as a screen for immune cells that were significantly different between OSCC and controls (*p*‐value < 0.05). Then, the “corrplot” (version 0.92) served to evaluate the connection between prognostic genes and differential immune cells (Saidak et al. 2021).

### 2.9 Correlation Analysis Between Risk Score and Immunotherapy

Immunological checkpoints with substantial variations between risk groups were tested on the TCGA‐OSCC dataset. Differences in tumor mutational load (TMB) between risk groups were determined by the Wilcoxon test, and the correlation between risk scores and TMB was calculated in conjunction with Spearman analysis. Subsequently, Tumor Immune Dysfunction and Exclusion (TIDE) scores were calculated on the TIDE website (http://tide.dfci.harvard.edu/) for the risk groups, and differences between groups were calculated using the Wilcoxon test.

### 2.10 Analysis of Drug Sensitivity

The 138 targeted therapeutic agents for OSCC on the Genomics of Drug Sensitivity in Cancer (GDSC) (https://www.cancerrxgene.org/) were first acquired. The 50% inhibitory concentration (IC50) of drugs acting on OSCC was then determined in combination with the “pRRophetic” (version 0.5) (Geeleher et al. 2014). Finally, the IC50 of drugs acting on OSCC was calculated using the “oncoPredic” (version 0.1), and differential expression was calculated by the Wilcoxon test (Maeser et al. 2021).

### 2.11 Expression Validation of Prognostic Genes

To conduct an initial investigation into the expression differences of prognostic genes between OSCC samples and matched paracancerous controls, we first performed a small‐scale validation of the bioinformatics findings. A total of five paired tumor and paracancerous samples were collected from the First Affiliated Hospital of Xinjiang Medical University (Affiliated Stomatological Hospital). The clinical characteristics of these 10 samples are provided in Table S1. Written informed consent was obtained from all participants, and the study protocol was approved by the Ethics Committee of the First Affiliated Hospital of Xinjiang Medical University (Approval No. K2023‐06‐04). This validation cohort is relatively small and serves only as a preliminary proof‐of‐concept. First, total RNA was extracted from all samples using TRIzol reagent (Ambion, Shanghai, China). Subsequently, RNA concentrations were measured by NanoPhotometer N50. Second, mRNA was reverse transcribed to synthesize cDNA using the SweScript First Strand cDNA synthesis kit (Servicebio, Wuhan, China). RT‐qPCR was conducted to assess the mRNA expression levels of prognostic genes. The amplification reaction conditions were as follows: initial denaturation (95°C for 1 min), denaturation (95°C for 20 s), annealing (55°C for 20 s), and extension (72°C for 30 s), and this was followed by the execution of 40 cycles. The relative expression levels of mRNA were calculated by the 2^−ΔΔCT^ method with GAPDH as an internal reference (Rao et al. 2013). The primer sequences used in the experiment are displayed in Table 1.

**Table 1:** Primers used for reverse transcription‐quantitative PCR analysis.

Primers	Sequences	NCBI gene ID	PCR product (bp)
ALB	F: 5‘‐ ACCTAGGAAAAGTGGGCAGC ‐3’	213	183
	R: 5‘‐ CTGAAAAGCATGGTCGCCTG −3’		
PDK42	F: 5‘‐ CGGCTTGCCAATTTCTCGTC −3’	5166	222
	R: 5‘‐ GCCAGGTTCTTTGGTTCCCT −3’		
SERPINA5	F: 5‘‐ TGTGGCAAAGCAAACGAAGG −3'	5104	165
	R: 5‘‐ ACAGTCTCCGAGGTCACGTA −3’		
CGNL1	F: 5‘‐ AAGCTGCCGAGTAAAGTGCT −3’	84952	112
	R: 5‘‐ CGGTGCTGTCCTTGGAGAAT −3’		
IL17F	F :5‘‐ GAGCCTTTGGACCGCTGAAC −3’	112744	106
	R:5‘‐ CGCTGGTGGCTTACTTTGTG −3’		
GAPDH	F :5‘‐ CGAAGGTGGAGTCAACGGATTT −3’	2597	148
	R:5‘‐ ATGGGTGGAATCATATTGGAAC −3’		

### 2.12 Statistical Analysis

The R programming language (version 4.2.2) was used for all analyses, and the Wilcoxon test was employed to evaluate the data from various groups. A *p*‐value of less than 0.05 was deemed statistically significant, unless otherwise noted.

## 3 Results

### 3.1 Determination of 263 DE‐NFATRGs

**Figure 1:**
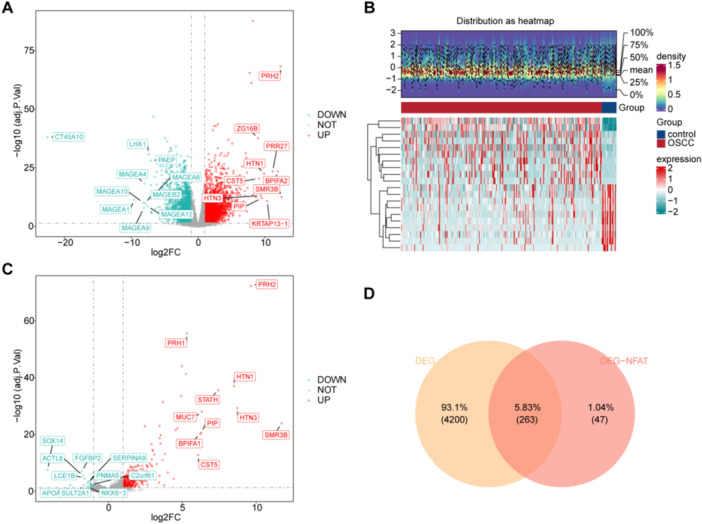
Differentially expressed genes between the OSCC samples and control samples. (A) Volcano plot of differential gene analysis. Red dots represent upregulated genes, green dots represent downregulated genes, and gray dots represent genes with no significant difference or small fold changes. (B) Heatmap of differential gene analysis. The upper half displays a heatmap of expression density, while the lower half shows the expression heatmap. (C) Volcano plot of DEG‐NFATs analysis. Red dots represent upregulated genes, green dots represent downregulated genes, and gray dots represent genes with no significant difference or small fold changes. (D) The Venn diagram of DEGs and DEG‐NFATs.

In the TCGA‐OSCC dataset, altogether 4463 DEGs were identified between OSCC samples and control samples, of which 2332 were up‐regulated and 2131 were down‐regulated (Figure 1A). The DEGs between groups were visualized by a heatmap (Figure 1B). A total of 310 DEG‐NFATs were identified between OSCC samples and control samples, of which 287 were up‐regulated, and 23 were down‐regulated (Figure 1C). The intersection of DEGs and DEG‐NFATs yielded a grand total of 263 DE‐NFATRGs. (Figure 1D).

### 3.2 Functional Enrichment Analysis and PPI Network of 263 DE‐NFATRGs

**Figure 2:**
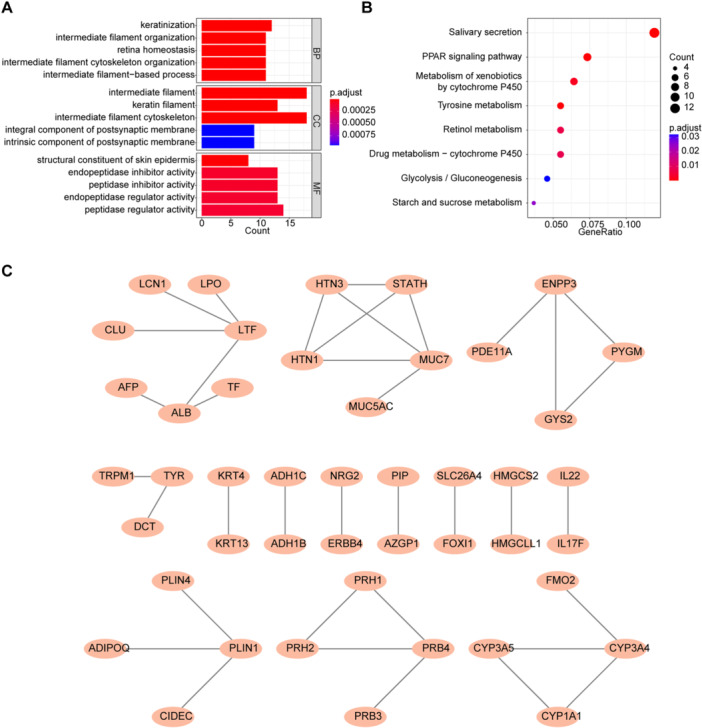
Functional enrichment analysis and PPI network of 263 DE‐NFATRGs. (A) GO functional analysis of DE‐NFATRGs. (B) KEGG pathways analysis of DE‐NFATRGs. (C) PPI network of DE‐NFATRGs.

DE‐NFATRGs were enriched for biological functions such as keratinization, keratin filaments, and endopeptidase inhibitor activity (Figure 2A). And they were mainly clustered on such as the PPAR signaling pathway, drug metabolism‐cytochrome P450, and tyrosine metabolism (Figure 2B). Among the 263 DE‐NFATRGs, ALB interacted strongly with AFP, TF, and LTE. There was a strong correlation between IL17F and IL22 (Figure 2C).

### 3.3 Determined of Seven NFAT‐Related Prognostic Genes

**Figure 3:**
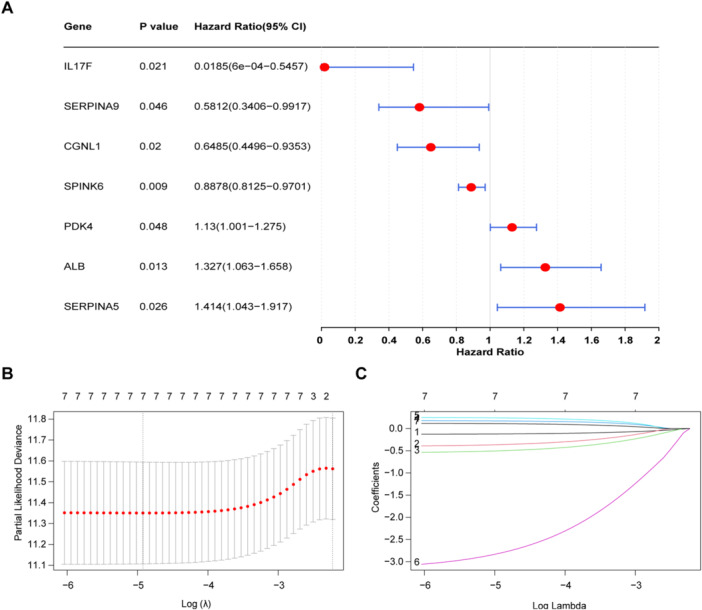
Determined of seven NFAT‐related prognostic genes. (A) Univariate Cox proportional hazards analysis. (B, C) Lasso regression analysis.

Seven prognostic‐related genes were obtained by univariate Cox regression analysis (Figure 3A). Lasso regression analysis was further performed on seven prognosis‐related genes, and SPINK6, SERPINA5, SERPINA9, CGNL1, ALB, IL17F, and PDK4 were all selected (Figure 3B,C).

### 3.4 Risk Model and Validation of OSCC

**Figure 4:**
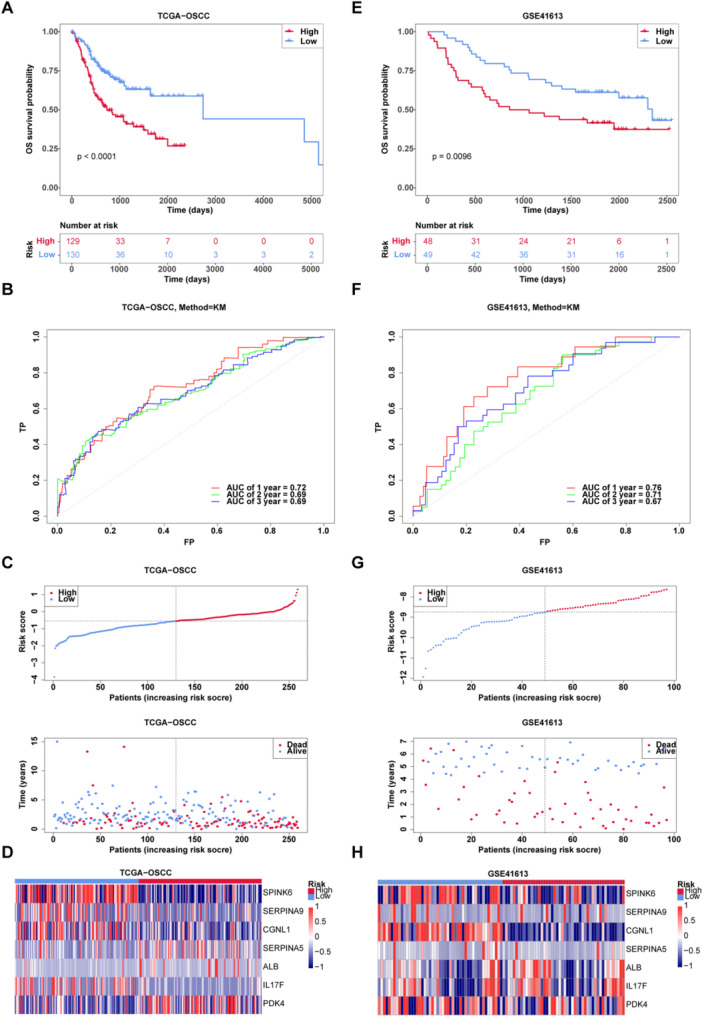
Risk model construction and validation. (A) Kaplan–Meier survival curve for TCGA‐OSCC. (B) ROC curve validating the ability to accurately predict TCGA‐OSCC survival. (C) Risk scores and survival curves for high‐ and low‐risk groups. (D) Gene expression patterns between high‐ and low‐risk groups. (E–H) Validation results from the GSE41613 dataset.

The median risk score was used as the cutoff to divide OSCC patients into high‐risk and low‐risk groups. Survival curves showed that the survival rate of the high‐risk group was much lower than that of the low‐risk group, and there was a statistically significant difference between the two (*p*‐value < 0.05) (Figure 4A). The areas under the ROC curves demonstrated modest discriminative ability (AUC > 0.6), indicating that the risk model has preliminary utility in predicting survival rates of OSCC patients. However, according to common standards (AUC > 0.7 for good performance), the model's discriminatory power is limited and requires further optimization (Figure 4B). The results from the risk curve graph indicated that as the risk score increased, the number of deceased cases also rose (Figure 4C). And the presence of differentially expressed NFAT‐related prognostic genes between different subgroups was confirmed (Figure 4D). The above results were consistent with those validated in the GSE41613 dataset (Figure 4E–H).

### 3.5 Analysis of the Clinicopathological Features and Prognosis of OSCC

**Figure 5:**
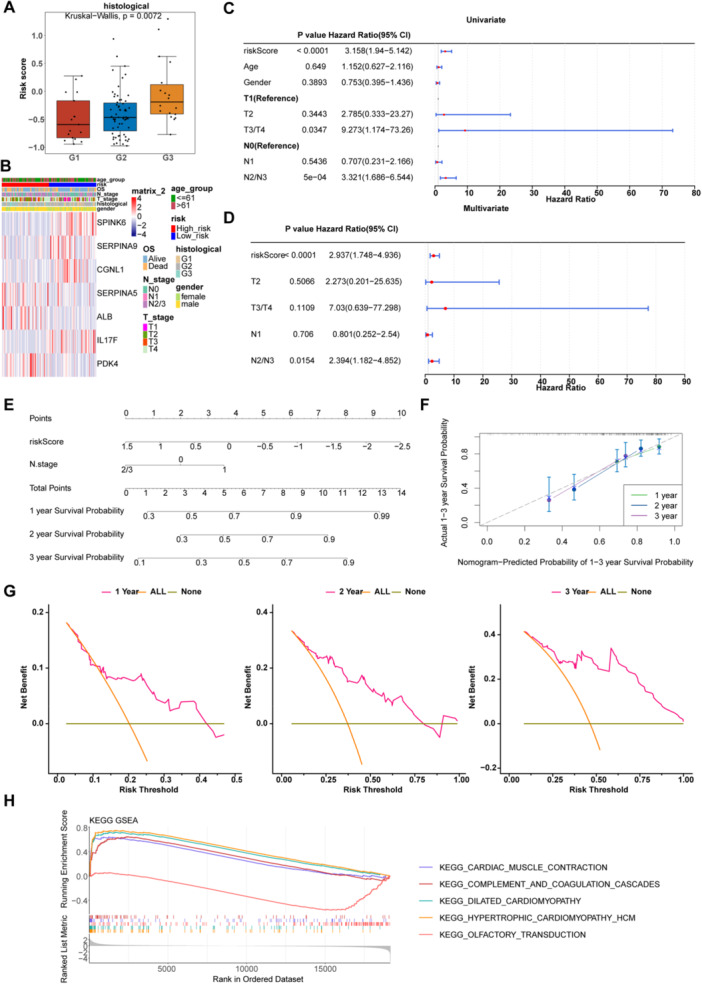
Analysis of the clinicopathological features and prognosis of TCGA‐OSCC. (A) The histologic risk score. (B) The seven NFAT‐related prognostic genes have different clinical features. (C, D) The TCGA‐OSCC survival prognosis strong correlation between the risk score and TN stage. (E) Nomogram was constructed by TCGA‐OSCC. (F) The calibration curve of the nomogram. (G) DCA curve of the nomogram. (H) The TCGA‐OSCC GSEA analysis.

Histologic grading for different pathological features (Figure 5A) was significantly different from the risk score (*p*‐value < 0.05). Expression of these seven NFAT‐related prognostic genes has different clinical features (Figure 5B). The OSCC survival prognosis showed a strong correlation (*p*‐value < 0.05) between the risk score and TN stage (Figure 5C,D). The rate of survival at 1 to 3 years for OSCC patients by the nomogram predicts (Figure 5E). The calibration curve confirmed that the nomogram had a high level of accuracy in predicting the survival for OSCC patients (Figure 5F). Furthermore, the DCA curves demonstrated the nomogram's clinical validity in for estimating OSCC patients’ chances of survival over a 3‐year period (Figure 5G). The KEGG analysis revealed that pathways like hypertrophic cardiomyopathy (HCM) and complement/coagulation cascades were significantly enriched in the high‐risk group compared to the low‐risk group (Figure 5H), suggesting their potential role in driving aggressive tumor behavior. This enrichment aligns with the GSEA findings and underscores the link between pathway activation and poor prognosis in OSCC.

### 3.6 Relationship Between Seven NFAT‐Related Prognostic Genes and Immune Cell Infiltration

**Figure 6:**
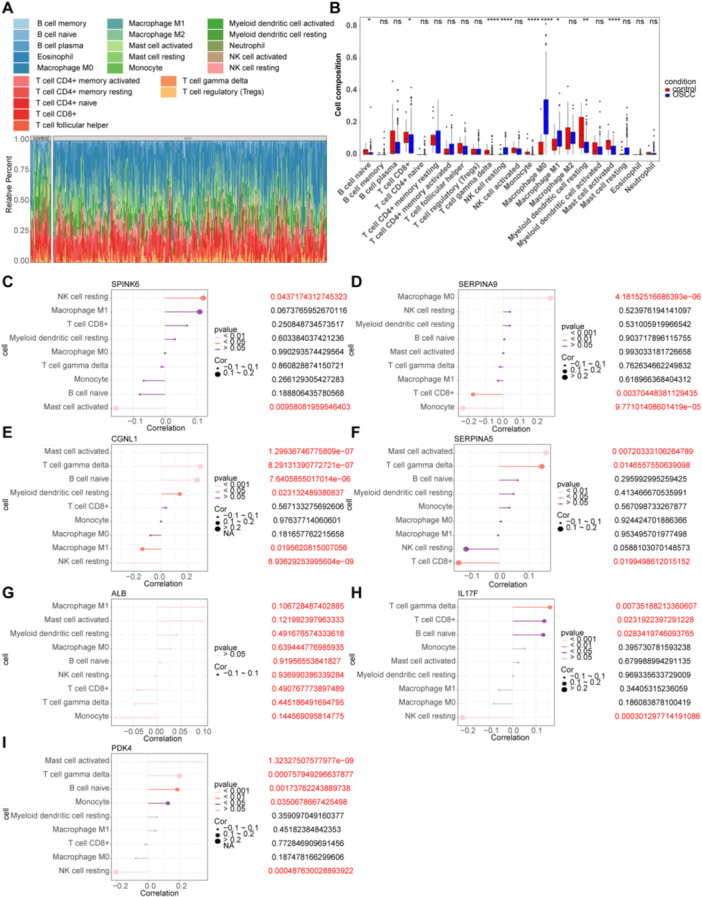
Relationship between seven NFAT‐related prognostic genes and immune cell infiltration. (A, B) Immune infiltration analysis of the expression heatmap of nine immune cells. (C–I) The seven NFAT‐related prognostic genes with these nine immune cells correlation analysis. (G) The ALB with nine immune cells correlated analysis.

The proportions of immune cell infiltration in the OSCC and control group are shown in Figure 6A. The results of the Wilcoxon test showed significant differences between the nine immune cells, such as naive B cells, CD8 + T cells, gamma delta T cells, and monocytes (Figure 6B). The correlation of seven NFAT‐related prognostic genes with these nine immune cells was demonstrated by lollipop charts (Figure 6C–I). Among them, the ALB was significantly correlated with all of these nine immune cells (Figure 6G).

### 3.7 Variations of Immunotherapy

**Figure 7:**
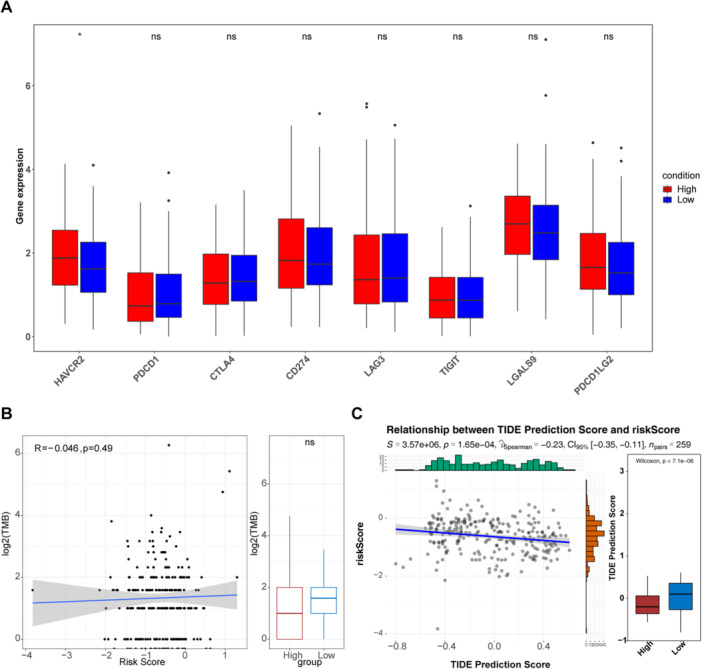
The variations of immunotherapy. (A) The HAVCR2 between the two groups correlation analysis. (B, C) The between the TIDE scores and the risk scores correlation analysis.

HAVCR2 was the most significant difference between the high‐risk and low‐risk groups (Figure 7A). There was no significant difference between TMB and risk scores (Figure 7B). There was a strong negative association between the TIDE scores and the risk scores, with the TIDE score being lower in the high‐risk group than in the low‐risk group (Figure 7C).

### 3.8 Drug Prediction for the Treatment of OSCC

**Figure 8:**
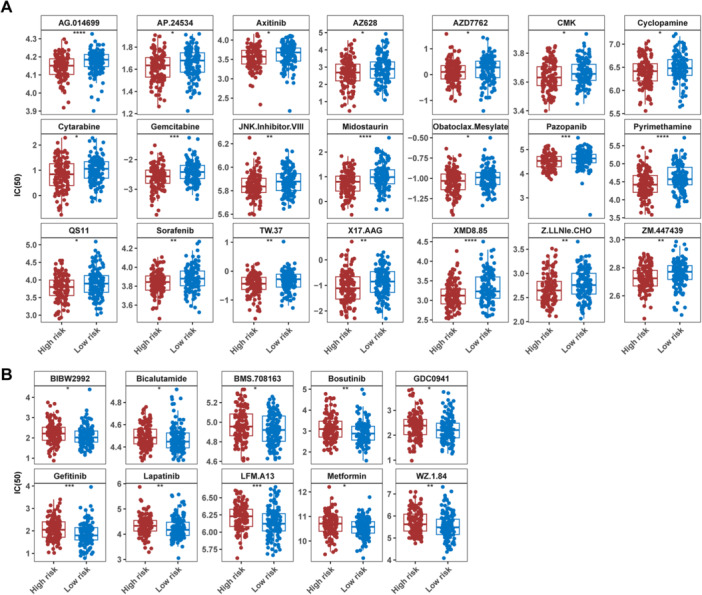
Drug prediction for the treatment of TCGA‐OSCC. (A) The 31 medications between the risk groups correlation analysis. (B) The 10 medications between the risk groups correlation analysis.

A total of 31 medications were found to differ significantly (*p*‐value < 0.05) between the risk groups, of which 21 had IC50 values that were lower in the low‐risk group, such as AG.014699, Midostaurin, Pyrimethamine, and XMD8.85 (Figure 8A). Conversely, the IC50 for 10 drugs was higher in the high‐risk group, such as BIBW2992, Gefitinib, and LFM.A13 (Figure 8B).

### 3.9 RT‐qPCR Analysis of Prognostic Genes Associated With NFAT

**Figure 9:**
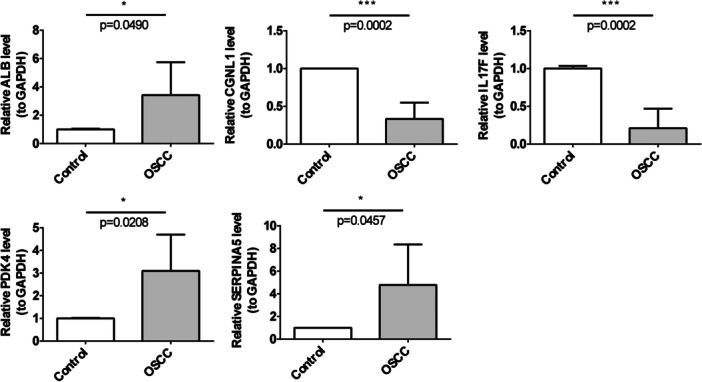
Differential expression analysis of prognostic genes in tumor tissues and paracancerous tissues.

Here, the clinical tissue samples were gathered to verify the difference in expression of prognostic genes between OSCC and control tissues. Given the limited sample size (5 paired samples), we randomly selected 5 genes (ALB, PDK4, SERPINA5, CGNL1, and IL17F) from the 7 prognostic genes for RT‐qPCR validation to ensure experimental efficiency within the constraints of this very small cohort. Among them, the OSCC group exhibited a significant up‐regulation in the mRNA expression of ALB, PDK4, and SERPINA5. In contrast, CGNL1 and IL17F displayed an inverse expression pattern, with its mRNA levels being significantly down‐regulated in the OSCC group (Figure 9).

## 4 Discussion

As one of the most common head and neck malignancies, OSCC ranks as the sixth most common cancer worldwide. The NFAT family of transcription factors, closely associated with OSCC, has not been fully elucidated in terms of its role in OSCC mechanisms. In this study, we identified seven NFAT‐related prognostic genes based on bioinformatics methods, including ALB (Albumin), PDK4 (Pyruvate Dehydrogenase Kinase 4), SERPINA5 (Serpin Family A Member 5), SPINK6 (Serpin Family A Member 6), SERPINA9 (Serpin Family A Member 9), CGNL1 (Cingulin Like 1), and IL17F (Interleukin 17F). Additionally, we delved into the potential mechanisms by which these seven genes participate in the development and progression of OSCC, which provided a theoretical basis for the prognosis and treatment of OSCC.

Serum ALB levels are one of the commonly used indicators for assessing patient malnutrition (Keller 2019). Research has shown that tumor cells in mice express a form of ALB that is distinct from the ALB found in normal serum. This tumor‐derived ALB has been observed to inhibit the activation, proliferation, and function of T cells both in vitro and in vivo, thereby promoting tumor growth. This finding suggests that tumor‐specific ALB may play a role in the immunosuppressive microenvironment of the tumor, contributing to the evasion of the immune system and the progression of cancer (Graner et al. 2004). Patient serum albumin levels are also one of the potential clinical indicators for predicting tumor prognosis. Chen et al. have previously developed the HALP (Hemoglobin, Albumin, Lymphocyte, and Platelet) score, which is based on the immune and nutritional status of cancer patients for predicting gastric cancer. Serum ALB level is a significant parameter in the calculation process of this score (Chen et al. 2015). Additionally, the C‐reactive protein‐to‐albumin ratio (CAR) and neutrophil percentage‐to‐albumin ratio (NPAR) have been identified as inflammation‐based parameters that can independently predict tumor prognosis in various types of cancer (Haruki et al. 2016; Kinoshita et al. 2015; Tawfik et al. 2016). Concurrently, studies have demonstrated that the all‐cause mortality rate in OSCC patients is inversely proportional to serum albumin concentration, suggesting a promising application prospect for these assessment methods in the treatment of OSCC (Bao et al. 2020).

PDK4 is one of the four isoforms of pyruvate dehydrogenase kinases that mediate the phosphorylation of pyruvate dehydrogenase (PDH). Other members of this kinase family include PDK1, PDK2, and PDK3 (Holness and Sugden 2003; Woolbright et al. 2019). It is well established that cancer cells can convert pyruvate to lactate through lactate dehydrogenase, thereby generating ATP through aerobic glycolysis, which promotes the proliferation of cancer cells (Stacpoole 2017). Phosphorylated PDH can inhibit the conversion of pyruvate to acetyl‐CoA, thereby facilitating this process (Sugden and Holness 2006). Consequently, pyruvate dehydrogenase kinases (PDKs) have garnered significant attention as therapeutic targets for cancer, and the investigation of PDK inhibitors has emerged as a focal point in research. For instance, Dong et al. (2016) have discovered that the PDK inhibitor diisopropylamine dichloroacetate (DADA) can induce the accumulation of reactive oxygen species (ROS) within cancer cells, thereby enhancing the radiotherapy sensitization of esophageal squamous cell carcinoma (ESCC). It is noteworthy that multiple studies have corroborated the significant association between PDK4 and the prognosis of oral squamous cell carcinoma (OSCC), with conclusions that are consistent with our research findings (Fan et al. 2022; Yue and Yao 2023).

SERPINA5 and SERPINA9 are both members of the serine protease inhibitor (Serpin) family. SERPINA5, specifically, is a physiological serine protease inhibitor of activated protein C (APC) and is one of the primary anticoagulant proteases in the human body (Suzuki et al. 1983). SERPINA5 plays a crucial role in preventing metastasis, inhibiting angiogenesis, and modulating inflammation in cancer (Jing et al. 2014). Multiple studies have indicated that SERPINA5 is associated with various types of cancer, including colorectal cancer, thyroid cancer, hepatocellular carcinoma, renal cell carcinoma, breast cancer, and ovarian cancer (Asanuma et al. 2007; Bijsmans et al. 2011; Brenner et al. 2013; Wakita et al. 2004; Zhang et al. 2021). Serine Peptidase Inhibitor Kazal Type 6 (SPINK6) is also a serine protease inhibitor that plays an important role in the regulation of epithelial‐mesenchymal transition (EMT), a key process in cancer cell proliferation, migration, and invasion (Thiery et al. 2009). Studies have shown that SPINK6 can enter the tumor microenvironment (TME) through autocrine and paracrine pathways, thereby facilitating the proliferation, invasion, and metastasis of cancer cells (Zheng et al. 2017). Although the precise molecular mechanisms by which SPINK6 mediates in OSCC have not been previously reported, it is postulated that its influence is extensive and spans the etiology and progression of OSCC, thereby presenting a broad scope for research and development.

CGNL1 is ubiquitously expressed in endothelial cells and serves as an inhibitor of RhoA activity at the zonula adherens and actin cytoskeleton junctions. It plays a significant role in embryonic development and angiogenesis processes (Chrifi et al. 2017; Citi et al. 2014; Guillemot et al. 2008). CGNL1 is associated with the prognosis of HNSCC patients harboring TP53 somatic mutations (Jin and Qin 2021). Furthermore, CGNL1 has significant correlations with endometrial cancer and High‐grade bladder urothelial carcinoma (HGBC) (Lu et al. 2022; Song et al. 2020). IL17F is a cytokine secreted by T cells, mast cells, and natural killer (NK) cells (Monin and Gaffen 2018). Immune infiltration analysis has revealed that IL17F is significantly associated with B naive cells, CD8 + T cells, gamma delta T cells, and resting NK cells, which is consistent with these studies. Several studies have demonstrated that IL17F can exert a protective effect on tumors such as colorectal cancer, OSCC, and liver cancer by influencing cell cycle and angiogenesis processes (Al‐Samadi et al. 2016; Almahmoudi et al. 2018; Ding et al. 2015; Xie et al. 2010).

We found that the complement and coagulation pathways are significantly associated with OSCC prognosis, which we hypothesize is mainly due to the strong effect of SERPINA5. The complement system plays a pivotal role in the pathogenesis and progression of cancer, and under specific circumstances, it exerts distinct functions (Afshar‐Kharghan 2017). Research has confirmed that patients with OSCC exhibit elevated concentrations of complement C3, complement factor B (CFB), and complement C4B in their saliva (Sivadasan et al. 2015). The coagulation pathway also exhibits a strong correlation with the occurrence and progression of OSCC. Studies have indicated that the coagulation system in OSCC patients possesses certain specificities, which are characterized by the overexpression of pro‐coagulant (F3) and pro‐fibrinolytic (PLAU) genes (Saidak et al. 2021).

According to the results of drug prediction analyses, it has been observed that the IC50 values for tyrosine kinase inhibitors, including bosutinib, gefitinib, and lapatinib, are elevated in the high‐risk stratum of OSCC patients relative to those in the low‐risk stratum. This suggests a reduced sensitivity or increased resistance to these agents among patients with a poorer prognosis. Research has substantiated that bosutinib possesses the capability to inhibit kinases beyond the Src family and can directly target the epidermal growth factor receptor (EGFR) for the treatment of HNSCC (Formisano et al. 2015; Lee et al. 2017; Nichols et al. 2014). Gefitinib, by virtue of its ability to inhibit the epidermal growth factor receptor (EGFR), effectively halts the proliferation, migration, and invasive capabilities of cancer cells. Its therapeutic efficacy has been well‐documented across a spectrum of malignancies, including non‐small cell lung cancer, HNSCC, colorectal carcinoma, and breast cancer, coupled with a favorable safety profile (Herbst et al. 2004; Ono and Kuwano 2006; Von Pawel 2004).

This study identified NFAT‐associated prognostic genes in OSCC and revealed promising targeted therapeutic agents based on the established prognostic model. However, the observed differential expression of these prognostic genes was derived from a limited sample size. Although the consecutive enrollment of samples without pre‐selection for specific clinical characteristics helps reduce selection bias, these findings should be regarded as preliminary validation. Therefore, the results require cautious interpretation. A sample size of five is sufficient for preliminary validation, but according to biostatistical principles, small sample sizes significantly reduce statistical power and increase the risk of false‐positive or false‐negative results. It is not reliable to detect minor but potential biological effects. Translating these discoveries into clinical applications will necessitate supplementary data from larger cohorts, validation through larger‐scale prospective studies, and further multi‐dimensional investigations. Interestingly, our study revealed a lower TIDE score in the high‐risk group, which theoretically suggests a better potential response to immunotherapy. However, this group exhibited a worse prognosis, creating an apparent discrepancy. This inconsistency may be attributed to the complexity of the tumor microenvironment in OSCC, where factors beyond TIDE—such as tumor mutational burden, immune cell exhaustion, or alternative immune evasion pathways—could dominate clinical outcomes. For instance, the high‐risk group might harbor additional immunosuppressive mechanisms that are not fully captured by TIDE scores, leading to poor survival despite a favorable TIDE profile. Future studies should validate TIDE's applicability in OSCC and explore integrated biomarkers to resolve this paradox. Additionally, our risk model was constructed using data from public databases, which may harbor inherent heterogeneity in patient selection, sample processing, and sequencing protocols. Moreover, to ensure cohort homogeneity, we specifically selected OSCC samples from certain anatomical subsites (e.g., oral cavity, buccal mucosa) from the TCGA dataset. While this approach enhances internal validity, it may limit the model's applicability to OSCC originating from other rare subsites and introduce potential selection bias. Finally, the disease data utilized in this study were primarily sourced from TCGA and GEO databases, which offer limited demographic and clinical background information. Consequently, the generalizability of this risk model across diverse ethnicities, geographical regions, and healthcare settings remains to be further evaluated. Future multi‐center international collaborative studies will be essential to validate the broad applicability of the model. We will continue to monitor subsequent research developments related to these genes and have initiated in‐depth investigations to elucidate their key molecular mechanisms in OSCC pathogenesis.

## Author Contributions

**Julaiti Tuerxun:** study design, data curation, statistics, formal analysis, writing of manuscript, review of manuscript. **Tairan Ding:** study design, review of manuscript. **Ailimaierdan Ainiwaer:** study design, data curation, statistics, formal analysis, review of manuscript.

## Funding

The authors have nothing to report.

## Ethics Statement

This study was approved by the ethics committee of the First Affiliated Hospital of Xinjiang Medical University (K2023‐06‐04) and was conducted in accordance with the Declaration of Helsinki. Each participant provided a written informed consent form at the time of registration, ensuring that all samples were collected in accordance with ethical guidelines.

## Consent

Written informed consent was obtained from all patients involved in this study.

## Conflicts of Interest

The authors declare no conflicts of interest.

## Supporting information


Supporting File 1

Supporting File 2

## Data Availability

The data that support the findings of this study are available on request from the corresponding author. The data are not publicly available due to privacy or ethical restrictions. Publicly available datasets were analyzed in this study, including data from the Genomic Data Commons (GDC, https://portal.gdc.cancer.gov/) and the Gene Expression Omnibus (GEO) database (accession number GSE41613). Additionally, the RT‐qPCR data generated from the 5 paired patient samples are available from the corresponding author upon reasonable request, in accordance with ethical approval (No. K2023‐06‐04).
