# Supplementary figures and images for: Identification of Prognostic Genes Relevant With the Nuclear Factors of Activated T Cells Based on Transcriptomics in Oral Squamous Cell Carcinoma
Source: Clin Exp Dent Res. 2026 Jul 19;12(4):e70408. doi: 10.1002/cre2.70408 (PMC13380819; doi:10.1002/cre2.70408)

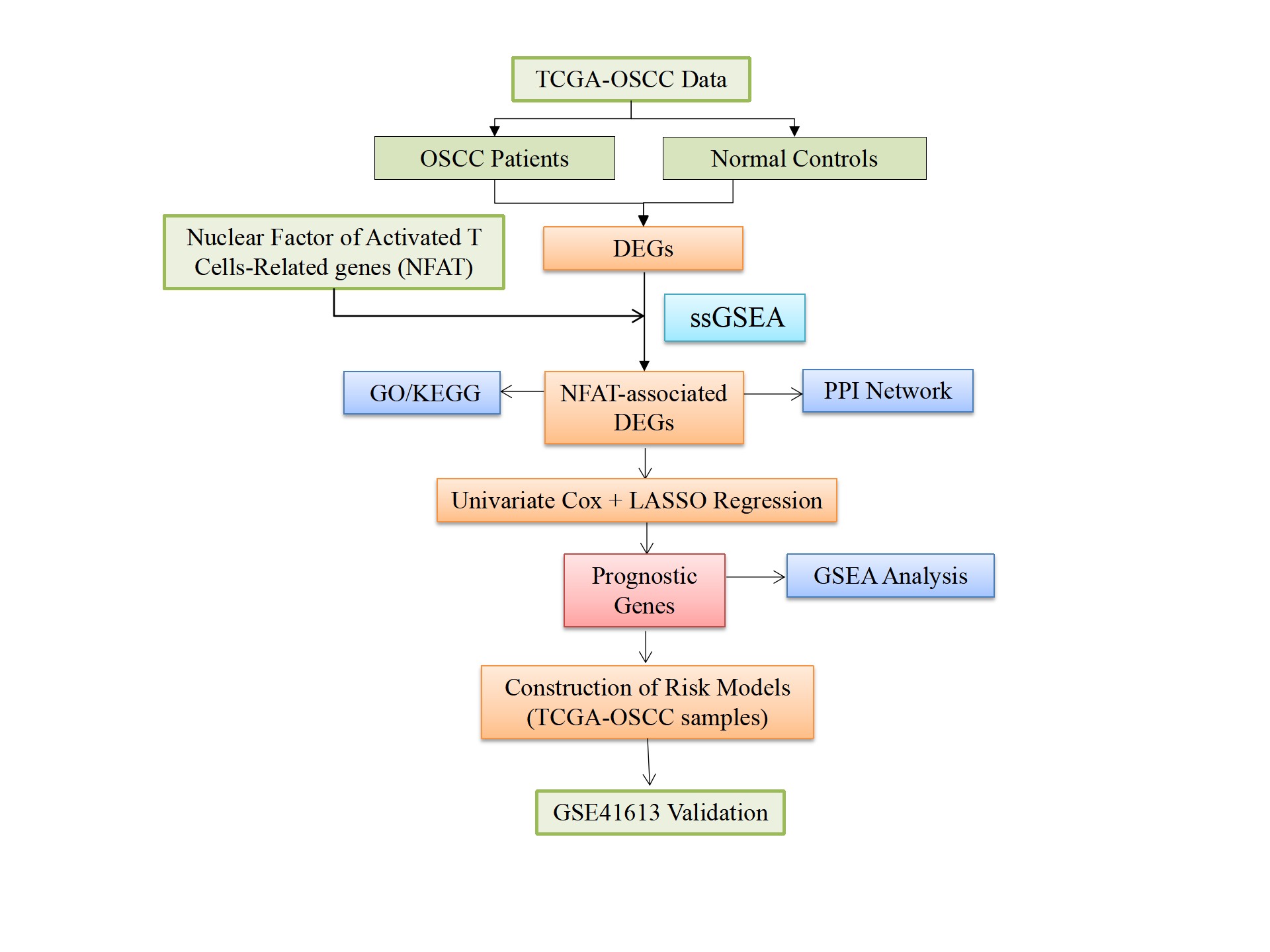

Supplement: Supplementary file 1 — Supporting File 1 [file CRE2-12-e70408-s001.tif]
